# Recurrence rates in Dupuytren’s contracture: a systematic review and meta-analysis comparing percutaneous and open surgical management^☆^

**DOI:** 10.1016/j.jpra.2026.10.004

**Published:** 2026-10-20

**Authors:** Funiba Ngwa, Alexander Chang, Alex Stratton-Moore, Gabrielle Knauer, Aryan Gupta, Natalia Mejia, Sarah Bauman, Sezgin Ciftci, Sameer Patel, Adam Walchak

**Affiliations:** a Lewis Katz School of Medicine at Temple University, Philadelphia, PA, USA; b Division of Plastic and Reconstructive Surgery, Lewis Katz School of Medicine at Temple University Hospital/Fox Chase Cancer Center, Philadelphia, PA, USA; c Charles E. Krausz Library, Temple University School of Podiatric Medicine, Philadelphia, PA, USA; d Department of Epidemiology and Biostatistics, Barnett College of Public Health at Temple University, Philadelphia, PA, USA

**Keywords:** Dupuytren’s contracture, Percutaneous needle fasciotomy, Fasciectomy, Recurrence, Meta-analysis, Systematic review

## Abstract

**Purpose:**

This systematic review and meta-analysis aims to compare pooled recurrence proportions following percutaneous versus open surgical treatment for Dupuytren’s contracture.

**Methods:**

A comprehensive search of PubMed, Embase, Cochrane Central, Web of Science, Science Direct, and CINAHL was conducted through January 2025 following PRISMA guidelines. Studies reporting recurrence rates after percutaneous or open surgical intervention for Dupuytren’s contracture were eligible for inclusion. Event proportions were pooled using logit transformation under fixed-effect and random-effects models. Heterogeneity was assessed using the I² statistic, Cochran's Q-test, and the likelihood ratio test. Subgroup analyses were performed by procedure type, and sensitivity analyses evaluated the influence of outlier studies.

**Results:**

Twenty studies met inclusion criteria. The overall pooled recurrence proportion under the random-effects model was 33.98% (95% CI: 24.98%–44.31%), with high between-study heterogeneity (I² = 94.8%). Subgroup analysis demonstrated a higher recurrence rate in the percutaneous group (39.5%; 95% CI: 26.3%–54.4%) compared to the open surgical group (24.4%; 95% CI: 18.5%–31.3%), with a statistically significant difference (p = 0.0472). Sensitivity analyses excluding influential studies yielded stable pooled estimates. No significant publication bias was identified in the percutaneous subgroup following exclusion of influential studies.

**Conclusions:**

Open surgical fasciectomy is associated with lower recurrence rates compared to percutaneous approaches for Dupuytren’s contracture, though recurrence remains substantial across both treatment modalities. Treatment selection should be individualized, incorporating disease severity, patient functional goals, and risk tolerance.

**Clinical relevance:**

These findings provide quantitative estimates of recurrence risk across treatment approaches and support individualized patient counseling. Clinicians should incorporate the higher recurrence burden associated with percutaneous techniques into shared decision-making discussions, particularly for patients weighing procedural invasiveness against long-term durability.

## Introduction

Dupuytren’s disease is a progressive fibroproliferative disorder affecting the palmar fascia, characterized by the formation of nodules and cords that result in involuntary flexion contracture of the digits, most commonly the ring and small fingers. These contractures can significantly impair hand function, limiting activities of daily living and diminishing quality of life.^1^ The condition predominantly affects individuals of Northern European descent, with prevalence increasing with age and a higher incidence among men.^2^ Genetic predisposition, advanced age, tobacco use, alcohol consumption, and diabetes mellitus have been identified as contributing risk factors.^3^ The clinical course varies considerably: for some patients the condition is mild and amenable to conservative management, whereas for others severe digital contracture necessitates operative intervention.

Among operative interventions, percutaneous needle fasciotomy (PNF) and other percutaneous techniques, including aponeurotomy, and open surgical fasciectomy represent the most widely employed approaches. Percutaneous needle fasciotomy is minimally invasive, performed under local anesthesia, and associated with shorter recovery time and lower cost relative to open surgery.^4^ However, its limited capacity to address the underlying diseased fascia raises concerns regarding elevated recurrence. Open surgical fasciectomy involves direct excision of the diseased palmar fascia, permitting more thorough removal of pathological connective tissue and more durable long-term outcomes,^5^ but carries greater invasiveness, prolonged recovery, and risks including wound infection, digital nerve injury, and postoperative stiffness.^1^ Additional modalities—collagenase clostridium histolyticum (CCH) injections, radiation therapy, and physical therapy—exist, but PNF and open fasciectomy remain the dominant operative choices.^6^, ^7^

Recurrence remains the pivotal outcome measure in Dupuytren’s management, as it determines long-term functional durability and likelihood of repeated intervention. For this review, recurrence is defined as the reappearance of clinically significant contracture requiring further intervention or producing meaningful impairment of hand function. Comparative evidence on recurrence rates between percutaneous and open approaches, particularly over extended follow-up, remains a subject of ongoing debate. This systematic review and meta-analysis were conducted to compare pooled recurrence proportions between percutaneous and open surgical treatments for Dupuytren’s contracture, providing clinicians with a quantitative framework to inform individualized treatment selection and patient counseling.

## Methods

### Search strategy

To identify eligible studies, the review team collaborated with an experienced medical librarian to develop comprehensive, database-specific search strategies. Search strategy development and reporting adhered to the PRISMA-S extension. The primary search was developed for PubMed and subsequently translated for all additional databases. The strategy was reviewed by the research team for accuracy and term relevance, and all finalized searches were peer-reviewed by an independent librarian in accordance with the Peer Review of Electronic Search Strategies (PRESS) checklist. No major limits or date restrictions were imposed.

The following databases were searched: PubMed (NLM), Embase (Elsevier), Cochrane Central Register of Controlled Trials (Wiley), Web of Science Core Collection (Clarivate), Science Direct (Elsevier), and CINAHL (EBSCOhost), using a combination of controlled vocabulary and free-text keywords. A grey literature search included ClinicalTrials.gov (www.clinicaltrials.gov) and the TRIP Medical Database (tripdatabase.com). All searches were executed on January 14, 2025. Full search strategies are archived at http://dx.doi.org/10.34944/dspace/10707.

The database search yielded the following results: PubMed (1809–01/14/2025): 1,364 records; Embase (1974–01/14/2025): 1,575 records; Cochrane Central (inception–01/14/2025): 106 records; Web of Science Core Collection (1900–01/14/2025): 263 records; Science Direct (inception–01/14/2025): 827 records; CINAHL (1976–01/14/2025): 142 records; ClinicalTrials.gov (inception–01/14/2025): 50 studies; TRIP Pro (1867–01/14/2025): 335 records.

### Study selection

The combined search retrieved 4,662 records. Following removal of 1,378 duplicate records using EndNote 20 and the Wichor deduplication method, 3,284 unique records were available for title and abstract screening, as illustrated in Fig. 1. Two blinded, independent reviewers performed title and abstract screening, followed by full-text review and final study selection. Discrepancies between reviewers were resolved through consensus or adjudication by a third independent reviewer.

**Fig. 1:**
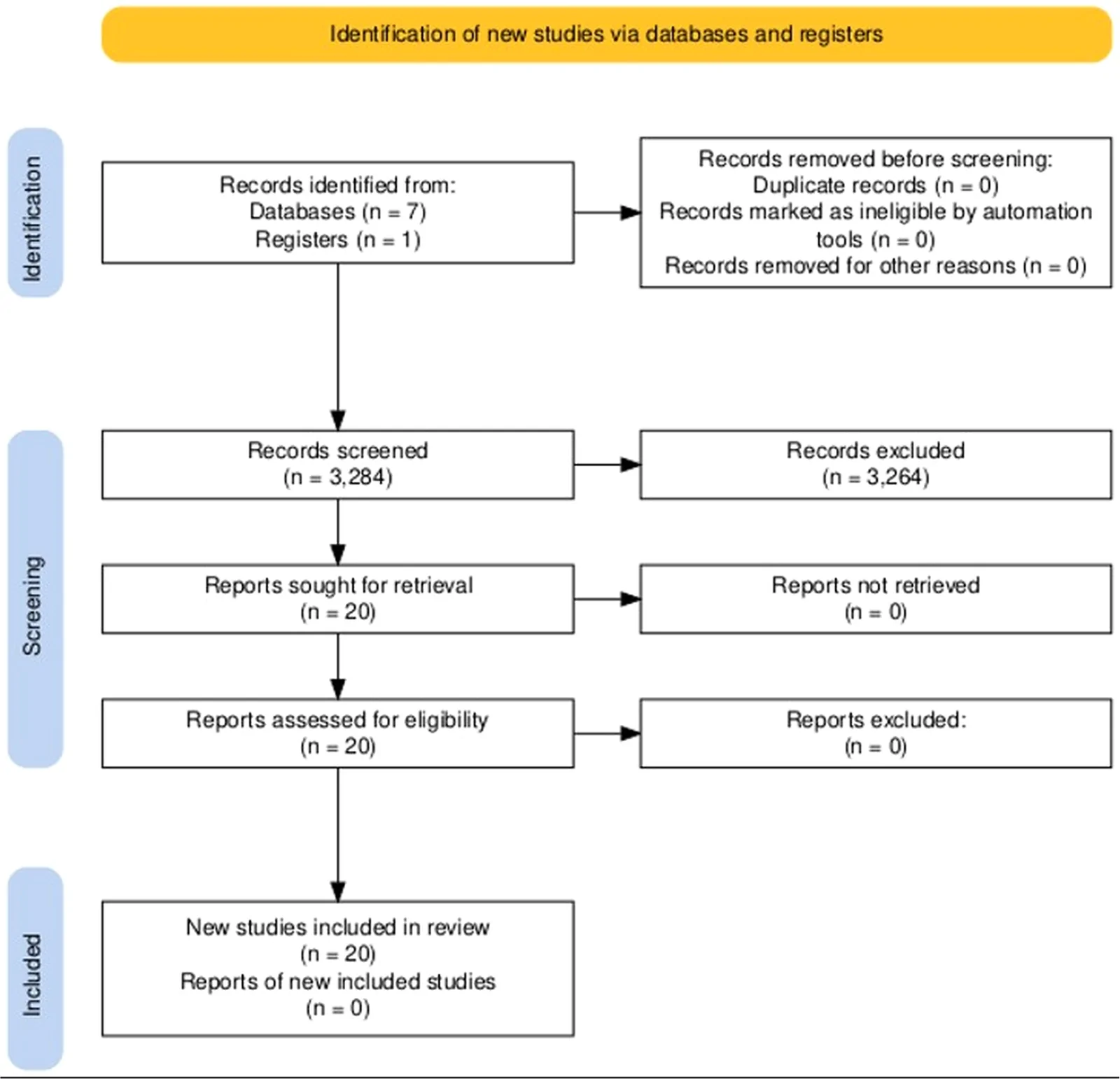
PRISMA flow diagram illustrating the systematic review screening and selection process.

### Statistical analysis

All statistical analyses were performed using R software (version 4.3.0 or later) with the “meta” and “dmetar” packages for meta-analysis of proportions. Event proportions were pooled using logit transformation to stabilize variance and approximate normality. Both fixed-effect (common-effect) and random-effects models (maximum likelihood estimator) were applied to estimate overall and subgroup-specific pooled proportions. Given the anticipated between-study variability, the random-effects model was designated as the primary analytic approach. Pooled estimates were back-transformed to proportions with 95% confidence intervals (CIs), and forest plots were generated to summarize findings.

Heterogeneity was quantified using the Cochran's Q-test and likelihood ratio test (LRT; significance threshold p < 0.05), the I² statistic (categorized as low [<25%], moderate [25%–50%], or high [>50%]), and tau² (between-study variance). Prediction intervals (95%) were calculated to characterize the expected range of true effects in future studies.

Subgroup analyses were conducted by procedure type (percutaneous vs. open) to evaluate differences in pooled recurrence proportions, with between-subgroup heterogeneity assessed using a mixed-effects model (significance threshold p < 0.05). Sensitivity analyses were performed using graphical outlier detection methods (Baujat plots), with pooled proportions re-estimated following exclusion of influential studies to evaluate the robustness of findings.

Publication bias was assessed using funnel plots for visual asymmetry and Egger’s linear regression test for quantitative evaluation (significance threshold p < 0.05). Formal testing was restricted to subgroups with a minimum of 10 studies (k.min = 10) to ensure adequate statistical power; findings from smaller subgroups were interpreted with appropriate caution.

Study-level recurrence proportions were logit-transformed and modeled by random-effects meta-regression with mean follow-up (months) as a single continuous predictor, fitted by restricted maximum likelihood with Knapp–Hartung adjustment of the standard errors. A linear form on the logit scale was pre-specified for interpretability and parsimony, given that the number of studies (14 percutaneous, 6 open) does not support estimation of an additional shape parameter; alternative log-time and complementary log-log specifications were examined and did not materially alter the estimates. Models were fitted separately within each treatment method and across all studies combined, and fitted recurrence was predicted at 12 and 60 months on the proportion scale, reported with 95% confidence intervals. One study (Anwar et al., 2007) did not report follow-up duration and was excluded, leaving 19 studies and 3,467 patients included in the meta-regression. Sample-size-weighted mean follow-up was 19.15 months for percutaneous, 32.24 months for open, and 26.00 months overall.

## Results

In the overall meta-analysis (Fig. 2), the common-effect model yielded a pooled event proportion of 0.2521 (95% CI: 0.2381–0.2666), whereas the random-effects model—based on a random intercept logistic regression with logit transformation—estimated a higher pooled proportion of 0.3398 (95% CI: 0.2498–0.4431). Heterogeneity was substantial, with tau² = 0.9465 (tau = 0.9729), I² = 94.8% (95% CI: 93.2%–96.0%), and H = 4.39 (95% CI: 3.84–5.02). Both Wald and likelihood ratio tests for heterogeneity were highly significant (Wald Q = 385.58, df = 20, p < 0.0001; LRT Q = 446.33, df = 20, p < 0.0001). Given the very high between-study variability, the random-effects model was selected as the preferred analytic framework. The analysis employed maximum-likelihood estimation for tau², logit transformation of proportions, and Clopper–Pearson confidence intervals for individual study estimates.

**Fig. 2:**
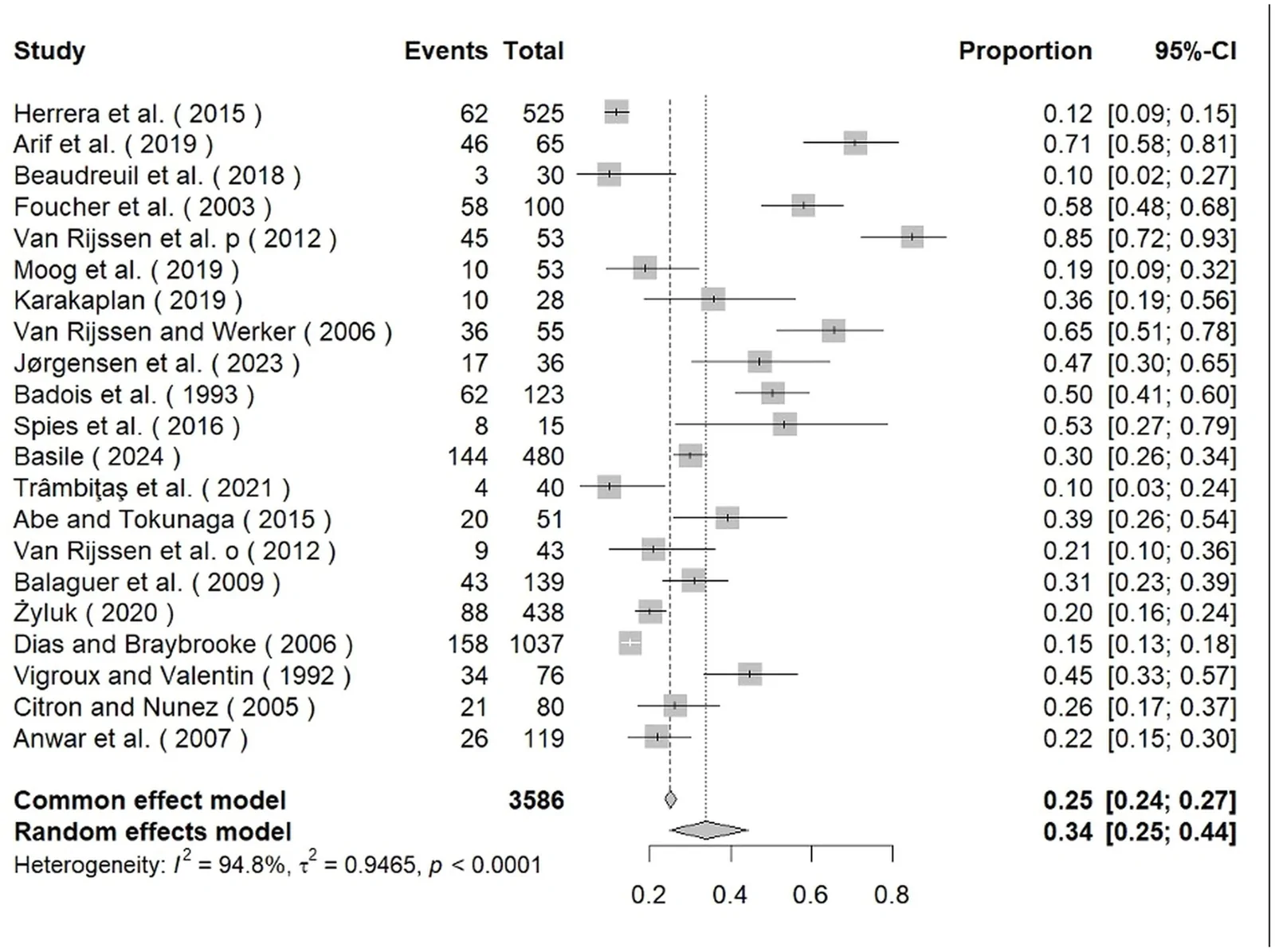
Forest plot for the overall meta-analysis of recurrence proportions. 20 total studies, with Van Rijssen et al. ^8^ counting twice because it contains open and percutaneous data^8^, ^9^, ^10^, ^11^, ^12^, ^13^, ^14^, ^15^, ^16^, ^17^, ^18^, ^19^, ^20^, ^21^, ^22^, ^23^, ^24^, ^25^, ^26^, ^27^.

Under the common-effect model (Fig. 3), percutaneous procedures demonstrated a pooled event proportion of 0.3174 (95% CI: 0.2954–0.3403; k = 14), with substantial heterogeneity (Q = 258.37, I² = 95.0%). Open procedures exhibited a lower pooled proportion of 0.1962 (95% CI: 0.1791–0.2145; k = 7), also with high heterogeneity (Q = 52.78, I² = 88.6%). The between-group comparison demonstrated a statistically significant difference (Q = 68.44, df = 1, p < 0.0001). Under the random-effects model, the pooled proportion for percutaneous procedures increased to 0.3949 (95% CI: 0.2629–0.5443; tau² = 1.2163), while open procedures yielded a pooled proportion of 0.2436 (95% CI: 0.1853–0.3134; tau² = 0.1743). The between-subgroup difference remained statistically significant, though attenuated after accounting for heterogeneity (Q = 3.94, df = 1, p = 0.0472). The random-effects framework was selected as the preferred approach given consistently high within-subgroup heterogeneity.

**Fig. 3:**
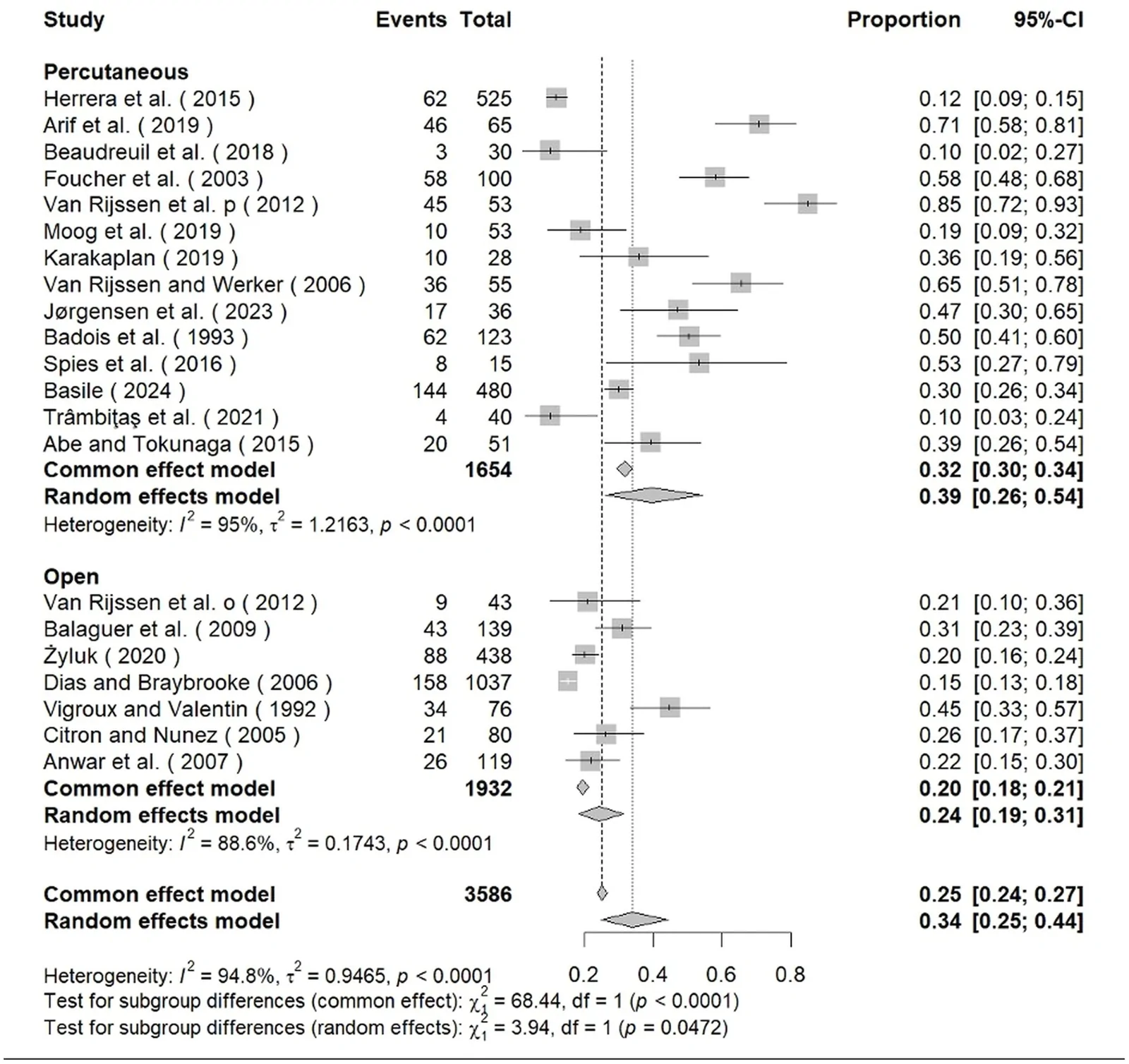
Forest plot for subgroup analysis stratified by procedure type (percutaneous vs. open)^8^, ^9^, ^10^, ^11^, ^12^, ^13^, ^14^, ^15^, ^16^, ^17^, ^18^, ^19^, ^20^, ^21^, ^22^, ^23^, ^24^, ^25^, ^26^, ^27^.

The random-effects model demonstrated an overall pooled recurrence proportion of approximately 34% (95% CI: 24.98%–44.31%), with very high between-study heterogeneity (I² = 94.8%). Subgroup analyses revealed substantially higher recurrence in the percutaneous group (39.5%) compared with the open surgical group (24.4%); this difference remained statistically significant under the random-effects model (p = 0.0472).

Heterogeneity within each subgroup remained high (I² > 88%), suggesting that additional moderating factors—such as patient characteristics, disease severity, operative technique variability, and inconsistent recurrence definitions—may contribute to outcome differences. Sensitivity analyses excluding influential studies (studies 1 and 18) reduced between-study heterogeneity and yielded stable pooled estimates, supporting the robustness of the primary findings.^13^^,^^22^ Assessment of publication bias identified no statistically significant asymmetry within the percutaneous subgroup based on Egger’s test (t = 1.17, p = 0.2662), although visual inspection of funnel plots suggested some asymmetry prior to exclusion of influential studies. The open surgical subgroup contained insufficient studies for reliable formal publication bias assessment. Following exclusion of influential studies, both Egger’s test (t = –0.73, p = 0.4779) and funnel plot assessment supported the absence of significant asymmetry in the percutaneous subgroup (Fig. 4). A Baujat plot was used to detect outliers and a forest plot was used to confirm that outliers were not significantly affecting the data (Fig. 5, Fig. 6).

**Fig. 4:**
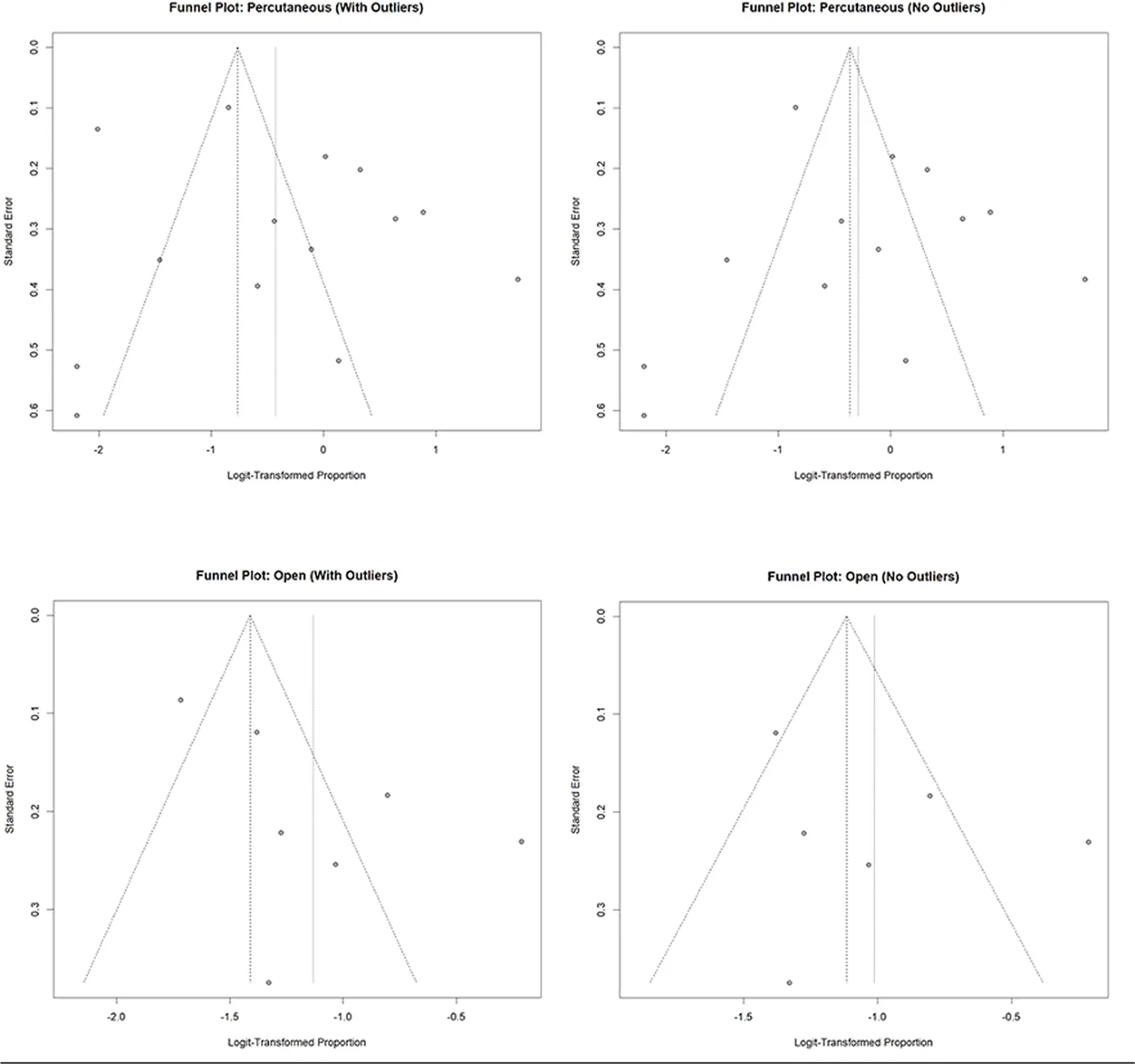
Funnel plots of percutaneous treatment and open treatment, with and without influential studies per procedural subgroup.

**Fig. 5:**
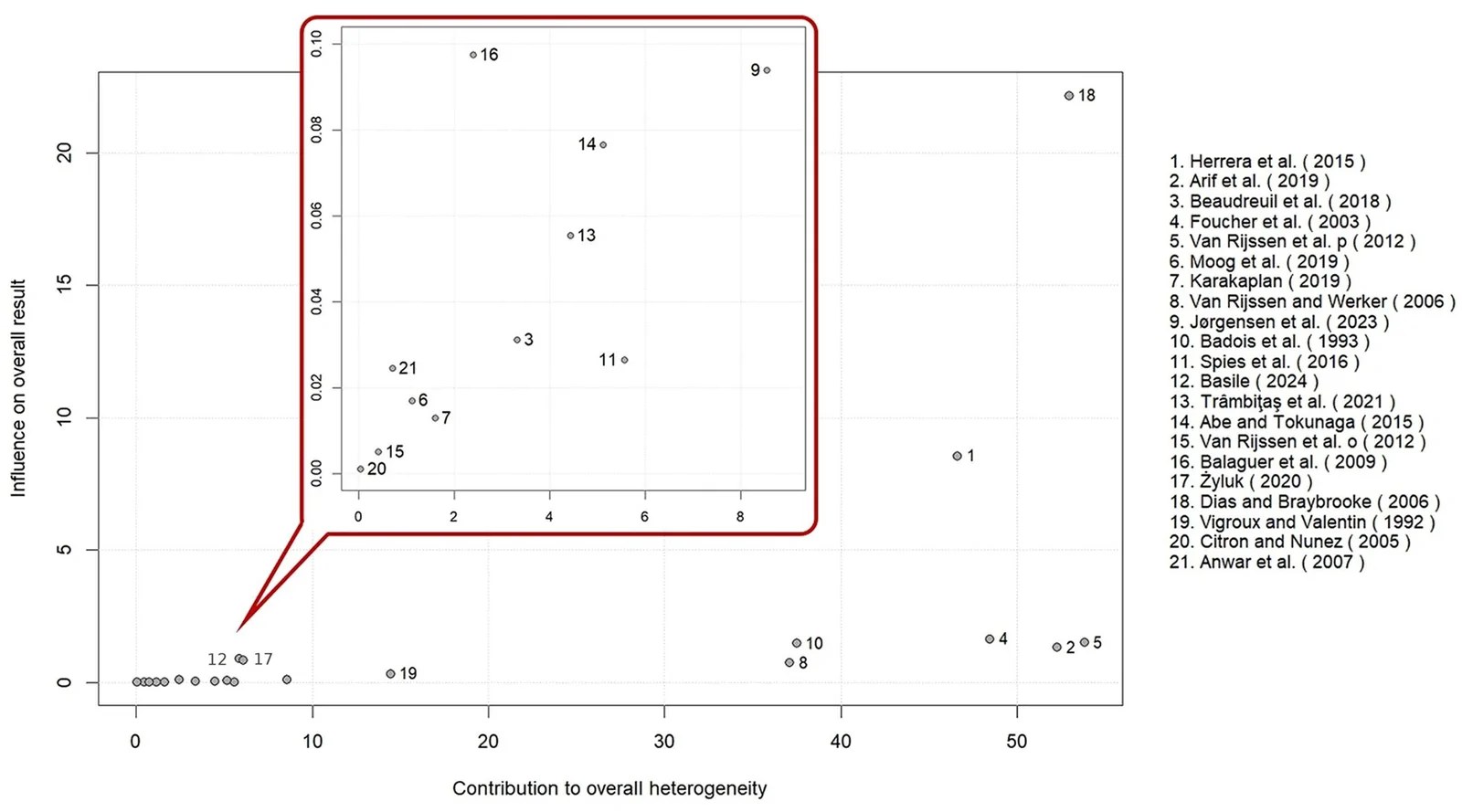
Baujat plot for detection of outlier studies.

**Fig. 6:**
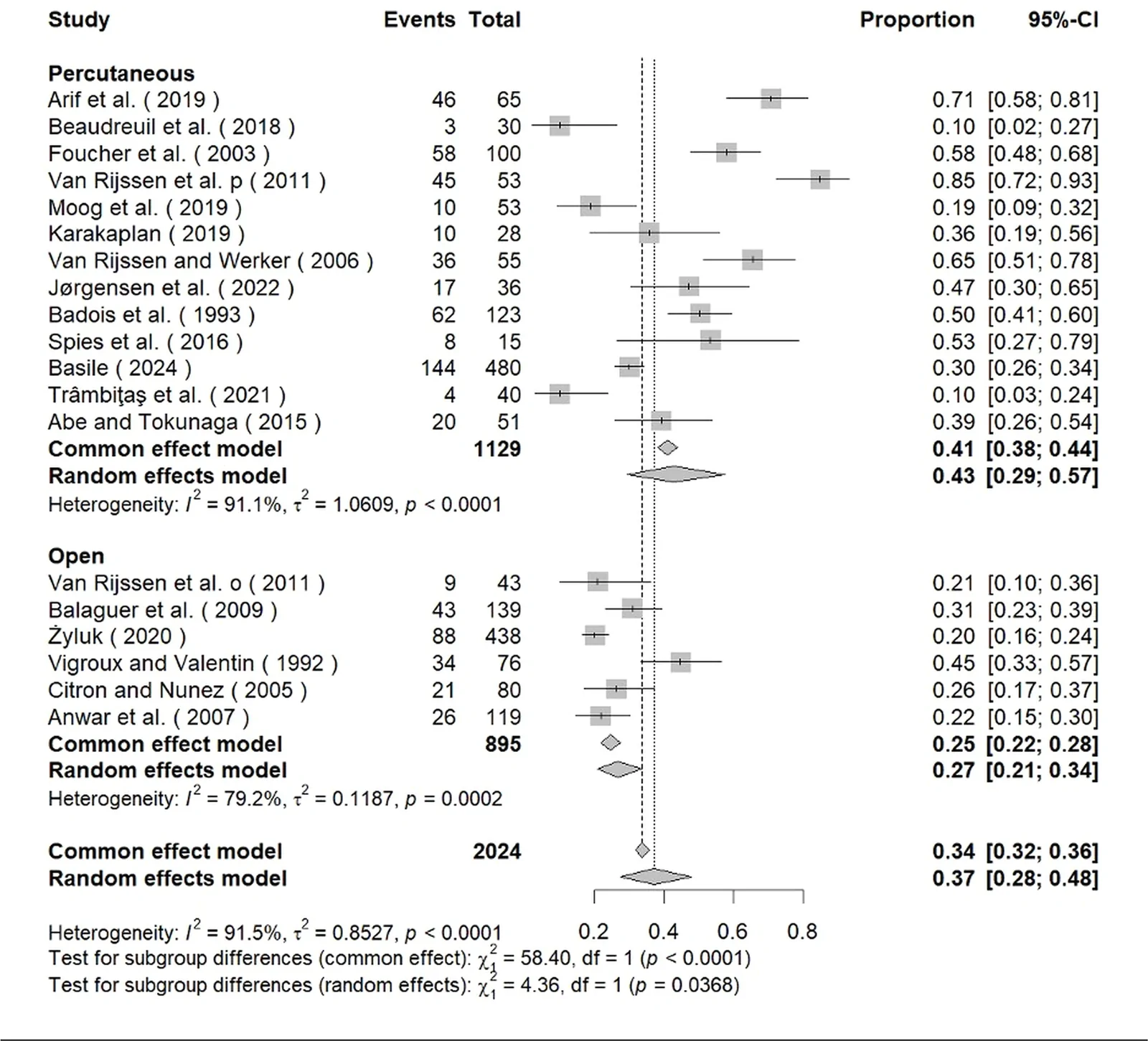
Forest plot following exclusion of outlier studies.

Recurrence increased with duration of follow-up in both methods, but the association reached conventional significance only for open surgery (odds ratio 1.12 per additional year, 95% CI 1.03-1.22; p = 0.031), where follow-up accounted for 71.0% of between-study heterogeneity. For percutaneous treatment, the estimated gradient was steeper but imprecise (OR 1.23 per year, 95% CI 0.83-1.82; p = 0.30) and explained only 3.3% of heterogeneity (residual tau-square = 1.20). Predicted recurrence at 1 and 5 years was 32.53% (95% CI 16.6-53.3) and 52.3% (95% CI 25.5-77.8) after percutaneous treatment, and 18.0% (95% CI 12.5-25.1) and 25.7% (95% CI 19.9-32.5) after open surgery (Fig. 7, Fig. 8). Prediction intervals were substantially wider at 5 years for both methods, therefore they were not reported.

**Fig. 7:**
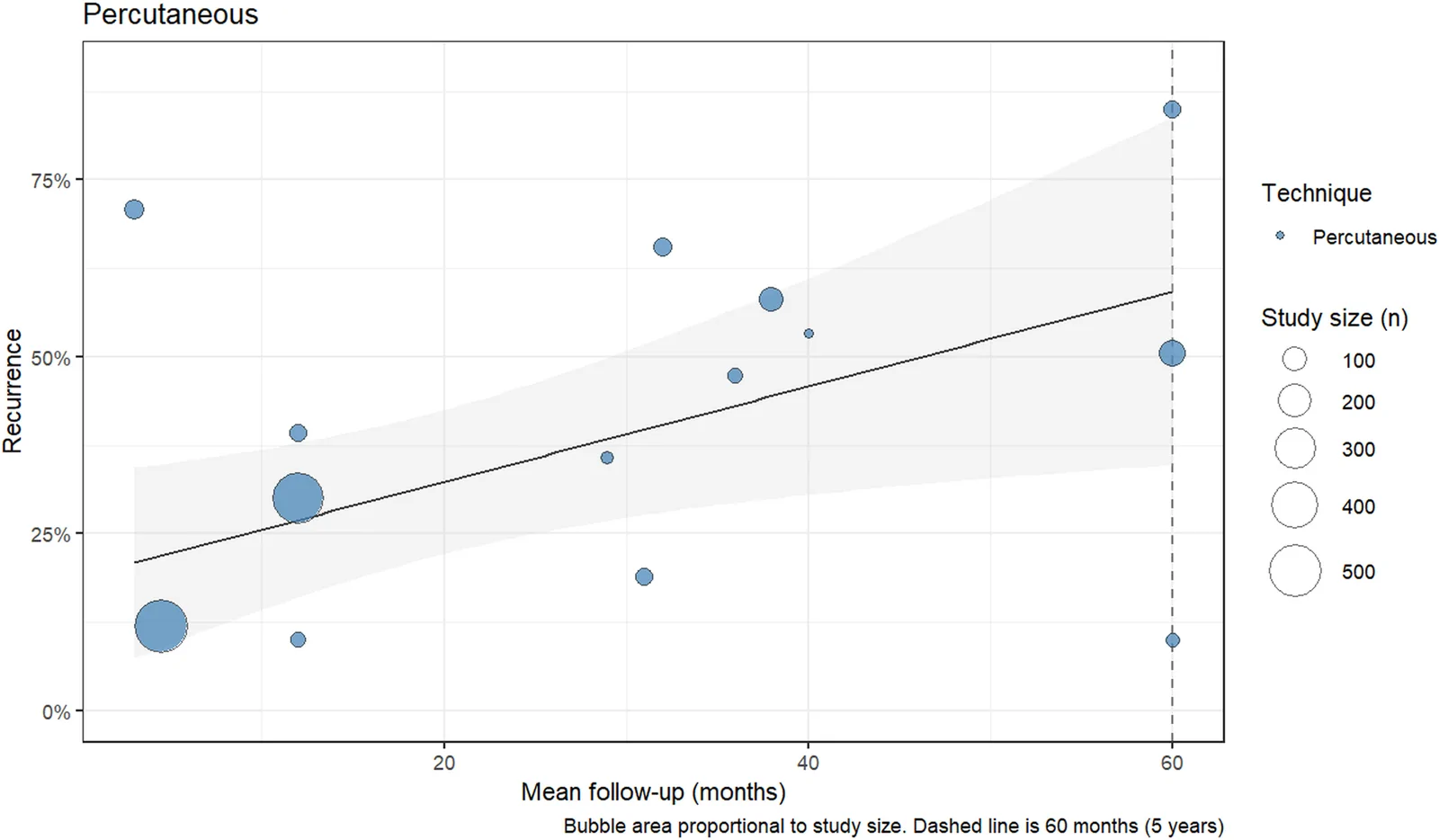
Bubble plot of recurrence rate against mean follow-up (months) for studies using the percutaneous technique, with bubble area proportional to study size (n) and the shaded band showing the 95% confidence interval of the fitted meta-regression line; the dashed vertical line marks 60 months (5 years).

**Fig. 8:**
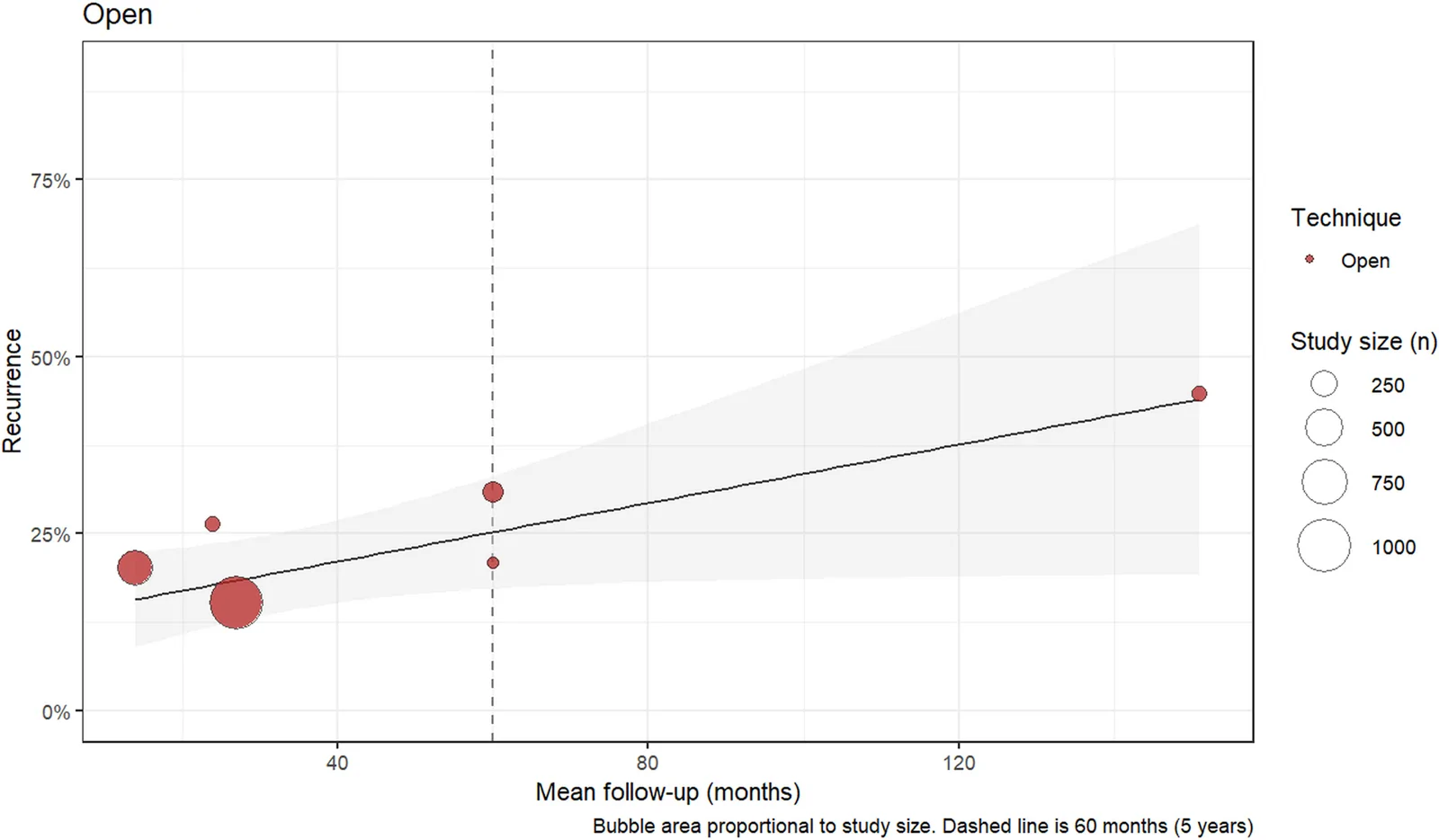
Bubble plot of recurrence rate against mean follow-up (months) for studies using the open technique, with bubble area proportional to study size (n) and the shaded band showing the 95% confidence interval of the fitted meta-regression line; the dashed vertical line marks 60 months (5 years).

## Discussion

This systematic review and meta-analysis evaluated recurrence following percutaneous and open surgical treatments for Dupuytren’s contracture, with recurrence defined as clinically meaningful contracture necessitating further intervention or resulting in functional impairment. Under a random-effects framework, the pooled recurrence proportion was approximately 34%, reflecting substantial between-study heterogeneity (I² = 94.8%). When stratified by procedure type, percutaneous treatments demonstrated a higher pooled recurrence proportion (39.5%) compared with open surgical procedures (24.4%), and this difference remained statistically significant after accounting for between-study variability (p = 0.0472). These findings suggest a greater recurrence burden associated with percutaneous approaches, though interpretation is constrained by the degree of observed heterogeneity.

Notably, heterogeneity remained high within both procedural subgroups (I² > 88%), indicating that recurrence risk is influenced by factors beyond procedural category alone. Variability in patient selection criteria, disease severity at baseline, operative technique, recurrence definitions, and duration of follow-up likely contributed to this dispersion. While recurrence is inherently a time-dependent phenomenon, formal time-stratified subgroup analyses were precluded by inconsistent reporting of follow-up intervals and recurrence thresholds across included studies. Accordingly, interpretation of temporal durability relies on pooled estimates rather than standardized longitudinal comparisons.

The observed difference in pooled recurrence between percutaneous and open procedures is biologically plausible. Percutaneous techniques primarily mechanically disrupt pathological cords without resecting diseased fascia, potentially permitting continued fibroproliferative activity within residual tissue. In contrast, open surgical approaches facilitate more comprehensive excision of affected fascia, which may reduce the substrate available for recurrence, though disease progression is not entirely eliminated. Importantly, even among surgically treated cohorts, recurrence remained substantial, underscoring that no currently available intervention provides definitive disease eradication.

From a clinical perspective, these findings reinforce the importance of individualized treatment selection. Percutaneous approaches offer meaningful advantages including shorter recovery, reduced surgical morbidity, and lower upfront procedural burden, but are associated with higher pooled recurrence proportions. Open surgical treatments are associated with lower recurrence on average but involve greater procedural invasiveness and perioperative risk. The higher recurrence rates observed with PNF may necessitate repeated interventions over time, potentially offsetting initial cost and recovery advantages. Rather than establishing definitive procedural superiority, these findings highlight the fundamental trade-off between durability and invasiveness that should be incorporated into shared decision-making discussions with patients.

Given the degree of heterogeneity observed, recurrence estimates are more appropriately interpreted as probability distributions than as fixed values. Sensitivity analyses excluding influential studies yielded stable pooled estimates and reduced heterogeneity, supporting the robustness of the overall findings while highlighting the disproportionate influence of certain studies on aggregate results. Assessment of publication bias did not demonstrate statistically significant asymmetry within the percutaneous subgroup following exclusion of influential studies; however, the limited number of open-procedure studies precluded reliable formal bias testing for that subgroup. By carefully incorporating these considerations and integrating adjunctive therapies where appropriate, clinicians can tailor treatment strategies to individual patient needs and preferences, thereby optimizing outcomes in the management of Dupuytren’s contracture.

Several limitations warrant consideration. First, definitions of recurrence varied considerably across included studies, ranging from clinical examination thresholds to patient-reported outcomes and reintervention rates, introducing potential inconsistency in outcome ascertainment. Second, surgical techniques within both the percutaneous and open categories were heterogeneous, and few studies adjusted for known prognostic factors such as disease severity, digit involvement, or patient comorbidities. These limitations restrict causal inference and contribute to residual heterogeneity. Future research should prioritize standardized recurrence definitions, consistent reporting of follow-up duration, and incorporation of validated patient-reported outcome measures. Comparative studies evaluating combination strategies or sequential treatment interventions may further elucidate how to optimally balance durability with procedural burden.

## Conclusions

This systematic review and meta-analysis demonstrate that open surgical fasciectomy for Dupuytren’s contracture is associated with lower pooled recurrence rates compared with percutaneous treatment approaches, although recurrence remains common across both modalities. The more comprehensive resection of diseased fascia achieved through open surgery likely contributes to its comparatively lower recurrence rates. Percutaneous needle fasciotomy and related percutaneous techniques, while associated with higher recurrence, represent an important minimally invasive option for patients in whom open surgical intervention is less desirable or feasible, offering shorter recovery periods and reduced perioperative morbidity.

Treatment selection should ultimately be individualized, incorporating disease severity, patient functional goals, comorbidity burden, and risk tolerance. Adjunctive therapies, like physical therapy and collagenase clostridium histolyticum injections, may further enhance outcomes and mitigate recurrence risk, particularly among high-risk patients. The findings of this review underscore the need for further prospective research to standardize recurrence definitions, evaluate long-term outcomes across treatment modalities, and assess innovative approaches that balance procedural efficacy with invasiveness. Standardization of outcome measures, including objective contracture angle thresholds and validated patient-reported instruments, would substantially improve the comparability of treatment efficacy across future studies. Advancing our understanding of treatment outcomes and optimizing patient-centered care remain essential goals in the long-term management of Dupuytren’s contracture.

## Presented at

This work was previously presented at the American Association for Hand Surgery Annual Meeting on January 15, 2026, in Chula Vista, California, and at the Robert H. Ivy Pennsylvania Plastic Surgery Society 2025 Annual Meeting in Philadelphia, Pennsylvania.

## Funding and ethical approval

There was no funding for this project. As this was a systematic review, no IRB or other ethical approval was required for this study.

## Declaration of Competing Interests

All authors declare that they have no conflicts of interest in relation to this manuscript or the process of this study.
